# Lack of transparent reporting of trial monitoring approaches in randomised controlled trials: A systematic review of contemporary protocol papers

**DOI:** 10.1177/17407745221143449

**Published:** 2023-01-11

**Authors:** Shao-Fan Hsieh, Victoria Yorke-Edwards, Macey L Murray, Carlos Diaz-Montana, Sharon B Love, Matthew R Sydes

**Affiliations:** 1 MRC Clinical Trials Unit at UCL, Institute of Clinical Trials and Methodology, University College London, London, UK; 2 Division of Medicine, University College London, London, UK; 3 Health Data Research UK, London, UK; 4 NHS DigiTrials Programme, Data Services Directorate, NHS Digital, London, UK; 5 British Heart Foundation Data Science Centre, Health Data Research UK, London, UK

**Keywords:** Systematic review, trial monitoring, on-site monitoring, central monitoring, risk-based monitoring, reporting monitoring, protocol paper, randomised controlled trial

## Abstract

**Background:**

Monitoring is essential to ensure patient safety and data integrity in clinical trials as per Good Clinical Practice. The Standard Protocol Items: Recommendations for Interventional Trials Statement and its checklist guides authors to include monitoring in their protocols. We investigated how well monitoring was reported in published ‘protocol papers’ for contemporary randomised controlled trials.

**Methods:**

A systematic search was conducted in PubMed to identify eligible protocol papers published in selected journals between 1 January 2020 and 31 May 2020. Protocol papers were classified by whether they reported monitoring and, if so, by the details of monitoring. Data were summarised descriptively.

**Results:**

Of 811 protocol papers for randomised controlled trials, 386 (48%; 95% CI: 44%–51%) explicitly reported some monitoring information. Of these, 20% (77/386) reported monitoring information consistent with an on-site monitoring approach, and 39% (152/386) with central monitoring, 26% (101/386) with a mixed approach, while 14% (54/386) did not provide sufficient information to specify an approach. Only 8% (30/386) of randomised controlled trials reported complete details about all of scope, frequency and organisation of monitoring; frequency of monitoring was the least reported. However, 6% (25/386) of papers used the term ‘audit’ to describe ‘monitoring’.

**Discussion:**

Monitoring information was reported in only approximately half of the protocol papers. Suboptimal reporting of monitoring hinders the clinical community from having the full information on which to judge the validity of a trial and jeopardises the value of protocol papers and the credibility of the trial itself. Greater efforts are needed to promote the transparent reporting of monitoring to journal editors and authors.

## Background

Clinical trial monitoring^1^ plays a vital role in ensuring participants’ well-being is protected, trial data are accurate and complete, and the conduct of the trial complies with the protocol, regulatory requirements and Good Clinical Practice (GCP) guidelines.^2^‘Monitoring’ here refers to the methods used by a sponsor to oversee the conduct of, and reporting of data from, clinical trials, including the ‘review of site processes, procedures and records, and verification of the accuracy of data submitted to the sponsor’.^3^ In the past, clinical trials predominantly relied on on-site monitoring, in which trial monitors systematically and repeatedly visited sites to check processes and procedures and conduct source data verification. Central monitoring and risk-based monitoring have recently become more commonly used, in part attributable to the publication of guidelines on risk-based monitoring by the Food and Drug Administration and European Medicines Agency in 2013. The barriers to on-site monitoring during the COVID-19 pandemic have further reinforced this approach.^3–5^ In central monitoring, centrally collected data (such as that held in the trial database) are checked often to detect systematic errors, such as missing data, procedural errors or suspected fraud. It incurs relatively low costs.^6–10^ Risk-based monitoring has been endorsed by the International Council for Harmonisation of technical requirements for pharmaceuticals for human use,^2^ regulatory agencies,^3,4,11^ public–private partnerships^12^ and industry collaborations.^8,13^ In risk-based monitoring, sponsors conduct risk assessments designed to identify instances where participants’ safety and well-being might be at risk, or where there are risks to the integrity of the trial and its data. They then set out the appropriate monitoring approaches in the monitoring plan to mitigate those risks: these might comprise on-site monitoring, central monitoring or a mixed approach depending on those risks. The scope and frequency of this monitoring will also be defined according to the identified risks.^3,4,8,11,14^ Research has demonstrated that risk-based monitoring is not inferior to extensive on-site monitoring^15^ and has shown that the central monitoring of several metrics has prognostic value to identify new findings during on-site visits.^16^

‘Protocol papers’ are condensed versions of clinical trial protocols published in peer-reviewed journals to summarise key aspects of trials’ study design, conduct and plans for analysis.^17^ A growing literature recognises that protocol papers enable the clinical research community to better interpret and validate published results.^18,19^ Several collaborations have developed guidelines to promote the quality and completeness of trial reporting,^20,21^ and many journals have endorsed these guidelines for their publications. The SPIRIT (Standard Protocol Items: Recommendations for Interventional Trials) Initiative launched in 2007 and published a 2013 Statement which has become an international standard to improve protocol completeness. The SPIRIT Statement contains a checklist of 33 items recommended to form the minimum content of a clinical trial protocol and addresses quality assurance (e.g. procedures related to promoting data quality and auditing trial conduct) in clinical trial protocols and related documents.^20,22^ The Statement has gained wide endorsement among journals, regulators, funders, patient groups and those organisations conducting clinical trials,^23^ and is a good indication of the current consensus of what should be included in a protocol. One should anticipate that the information requested by SPIRIT should be included in a protocol paper.

In light of the critical role of monitoring in ensuring the quality and credibility of clinical trials, clear reporting of the monitoring approach chosen for a trial is essential. While surveys have studied the various monitoring approaches taken by organisations, there is little empirical research on how researchers report monitoring in protocol papers for public scrutiny.^24,25^ Moreover, since the SPIRIT Statement’s checklist recommends authors to include information on monitoring in protocols (Item 23 of the checklist), it is worthwhile finding out how far the SPIRIT Statement’s recommendation is being adhered to. In this systematic review, we aim to investigate how often monitoring is discussed and the type of monitoring details given in published protocol papers of randomised controlled trials (RCTs).

## Methods

### Search and eligibility criteria

A systematic article search was conducted via PubMed between April 2020 and June 2020 to identify recent RCT protocol papers. Due to the large number of protocol papers, the article publication dates were restricted to 1 January 2020 to 31 May 2020 to form a contemporary sample. A further restriction was made to limit the search to several key journals known by the authors to commonly publish substantial numbers of clinical trial protocol papers: *BMJ Open, Contemporary Clinical Trials, Contemporary Clinical Trials Communications, JMIR Research Protocols, Medicine (Baltimore), PLOS ONE* and *Trials*. We also included journals published by *BioMed Central (BMC)* as they also publish large numbers of protocol papers across their publications. However, as no individual journal in the publishing group’s portfolio was expected to publish a comparable number of protocol papers to the key journals, we chose to analyse them together as a group.

The search terms used were ‘protocol’ AND ‘(randomized OR randomised)’ filtered by the publication date and key journals (Supplementary file 1). Protocol paper titles and abstracts were screened for eligibility, and those eligible were retrieved and underwent a full-text data extraction.

### Inclusion criteria

Protocol papers were included where they were published in *BMJ Open, Contemporary Clinical Trials, Contemporary Clinical Trials Communications, JMIR Research Protocols, Medicine (Baltimore), PLOS ONE, Trials and BMC-series* journals, and published between 1 January and 31 May 2020.

### Exclusion criteria

We excluded papers detailing observational studies and non-randomised trials, systematic reviews or meta-analyses of RCTs, trial results of RCTs, and others (e.g. non-human studies, methodological studies, correction letters). We also excluded feasibility trials collecting monitoring findings as a primary or secondary endpoint.

### Definitions of monitoring

Data describing the monitoring were extracted by reviewing the full paper using pre-determined classification criteria for what constitutes and does not constitute ‘monitoring’ (the methods used by a sponsor to oversee the conduct of, and reporting of data from, clinical trials). All monitoring activities carried out either by sponsors or their delegates in the form of on-site monitoring, central monitoring or in a mixed approach (in which both on-site and central monitoring are utilised) were considered ‘monitoring’. We note that any of these methods may be determined by a risk assessment in risk-based monitoring, although a mixed approach may be anticipated, as in risk-based monitoring, the monitoring methods are tailored to each specific risk.

The following activities were not considered ‘monitoring’: monitoring activities carried out by ethics committees, those carried out by site investigators and their staff, and monitoring activities irrelevant to trial conduct (e.g. vital sign monitoring).

### Data extraction

Examples of how protocol papers mentioned monitoring information, and the corresponding classification of monitoring approaches, are given in Supplementary file 2. Descriptive data were extracted to summarise how often monitoring was reported in protocol papers in each key journal (calculated with a 95% confidence interval (95% CI)), and the way in which monitoring was reported.

Full data extraction was completed by one reviewer (S.-F.H.). The accuracy of data extraction and coding was double-checked in a random selection (5%) of protocol papers by five reviewers (M.R.S., S.B.L., M.L.M., V.Y.-E. and C.D.-M.). They also reviewed 5% of the excluded papers to confirm their exclusion. For papers containing monitoring information, six data items per paper (i.e. monitoring approach, monitoring organisation, scope and frequency of on-site monitoring, scope and frequency of central monitoring) were checked (120 data points in total). The extracted data were held in a Microsoft Excel spreadsheet (Microsoft, Redmond, Washington, USA).

### Analysis

Descriptive statistics were used to categorise and group the extracted information in Excel. Preferred Reporting Items for Systematic Reviews and Meta-Analyses (PRISMA) principles were followed for reporting (Supplementary file 3).^26^ The data were also analysed to compare those journals that endorsed or recommended the SPIRIT Statement in the submission guidelines on their website (*Trials, BMJ Open, Medicine (Baltimore), JMIR Research Protocols, PLOS ONE* and the *BMC* group of journals) with those who did not mention SPIRIT (*Contemporary Clinical Trials* and *Contemporary Clinical Trials Communications)*.^27–34^ As the SPIRIT Statement uses the term ‘auditing’ to denote ‘monitoring’, the data were also analysed to investigate the proportions of protocol papers using the term ‘auditing’ versus ‘monitoring’.^20,22^

### Result of double extraction for data checking

All reviewers agreed the sample of excluded papers should be excluded. The reviewers agreed the coding for all but four fields, for which they initially had discrepant interpretations of the original extraction (4/120, 3% of data points). These discrepancies were due to one reviewer missing critical words on monitoring that another reviewer identified. It was noted by all reviewers that most protocol papers reviewed did not provide a dedicated, structured paragraph to describe monitoring, and that this hampered data extraction. Those errors that were found occurred at random and the results from this review would not be improved by double data extraction and coding of the whole dataset. To be consistent throughout, the original data extraction by S.-F.H. was used.

## Results

**Figure 1.**
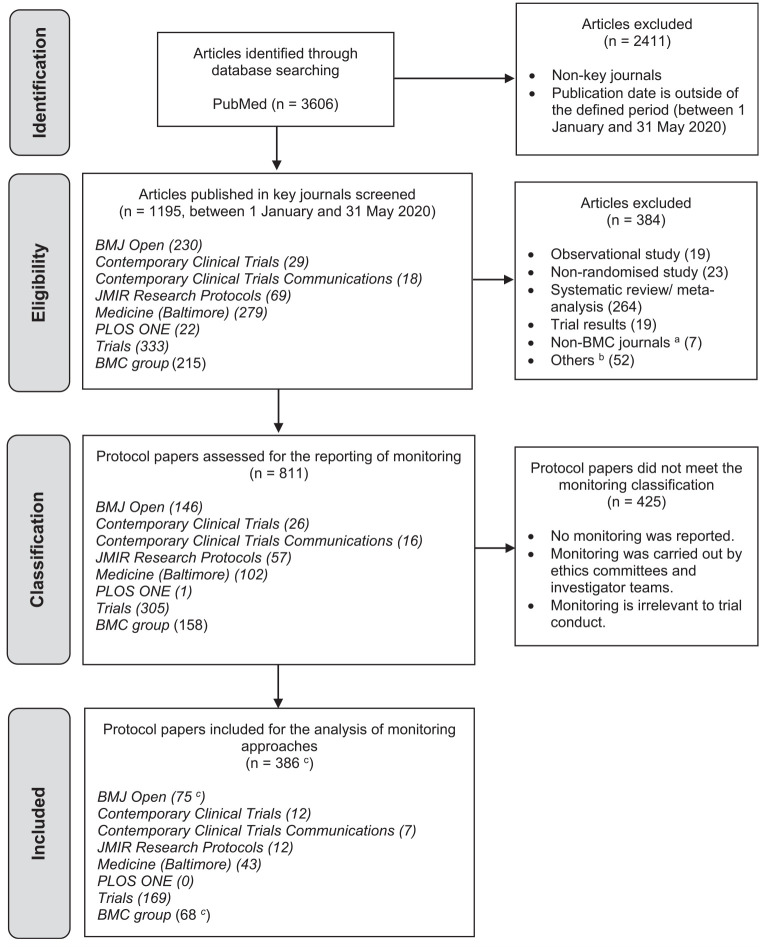
PRISMA flow diagram of article selection. ^a^The search term ‘BMC’ was used to identify BMC-series journals and yielded seven non-BMC-journal articles. ^b^Others including non-human studies, methodological studies, correction letters and feasibility trials collecting monitoring findings as a primary or secondary endpoint. ^c^This number included the two RCTs that explicitly reported that on-site monitoring was not needed (in BMJ Open and BMC group, respectively).

Figure 1 summarises the PRISMA flow diagram of article identification and selection, and a PRISMA checklist was completed (Supplementary file 3). The search yielded 1195 potential protocol papers. However, 384 articles were then excluded, with the majority of these excluded because they related to systematic reviews of RCT results (69%, 264/384) rather than RCTs directly. The remaining 811 were eligible protocol papers which then underwent data extraction.

### Reporting of monitoring and of monitoring details

Of the 811 eligible protocol papers describing RCTs, 386 (48%; 95% CI: 44%–51%) explicitly reported information about their monitoring approach, including two that offered justification that on-site monitoring was unnecessary because of the low-risk nature of the trials. How often monitoring was reported varied between journals from 21% to 55%. *Trials* and *BMJ Open* reported monitoring information in half of their protocol papers (at 55% and 51%, respectively), but *JMIR Research Protocols* only reported monitoring in less than a quarter of their protocol papers (Table 1).

**Table 1.** The reporting of monitoring and of monitoring approaches in protocol papers by journal endorsement/ recommendation of the SPIRIT statement.

Journal	Reporting of monitoring			Monitoring approaches		
	Monitoring reported /protocol papers	% (95% CI)	On-site % (n)	Central % (n)	Mixed % (n)	Unspecified % (n)
Journals endorsing/ recommending the SPIRIT guidelines						
Trials	169/305	55 (50, 61)	19 (32)	31 (52)	38 (65)	12 (20)
BMJ Open^a^	75/146	51 (43, 60)	26 (19)	30 (22)	24 (18)	20 (15)
BMC Group^a^	68/158	43 (35, 51)	18 (12)	63 (42)	9 (6)	10 (7)
Medicine (Baltimore)	43/102	42 (32, 52)	23 (10)	33 (14)	21 (9)	23 (10)
JMIR Research Protocols	12/57	21 (11, 34)	8 (1)	75 (9)	17 (2)	0 (0)
PLoS ONE	0/1	0 (0)	0 (0)	0 (0)	0 (0)	0 (0)
Subtotal	367/769	48 (44, 51)	20 (74)	38 (139)	28 (100)	14 (52)
Journals that do not mention the SPIRIT guidelines						
Contemporary Clinical Trials	12/26	46 (27, 67)	17 (2)	67 (8)	8 (1)	8 (1)
Contemporary Clinical Trials Communications	7/16	44 (20, 70)	14 (1)	72 (5)	0 (0)	14 (1)
Subtotal	19/42	45 (30,61)	16 (3)	68 (13)	5 (1)	11 (2)
Total	386/811	48 (44, 51)	20 (77)	40 (152)	26 (101)	14 (54)

SPIRIT: standard protocol items: recommendations for interventional trials; CI: confidence interval.

a Two RCTs (one publishing in BMJ Open, the other in a BMC group journal) explicitly reported on-site monitoring was not needed. These are not included in the reported pattern of monitoring approaches.

The SPIRIT Statement recommends the inclusion of trial monitoring details in protocols. Most of the journals in this review (*Trials, BMJ Open, Medicine (Baltimore), JMIR Research Protocols, PLOS ONE* and the *BMC* group of journals) endorse or recommend the SPIRIT Statement and 95% (769/811) of the protocol papers in this review were published in these journals. However, only 367 of these 769 (48%; 95% CI: 44%–51%) reported their monitoring approach.^27,28,31–34^ In protocol papers published in the other journals, the proportion was equally low, with 19 of the 42 (45%; 95% CI: 30%–61%) protocol papers reporting their monitoring approach (Table 1).

Of the 386 protocol papers that provided monitoring details, 20% (77/386) described an approach consistent with on-site monitoring, 39% (152/386) described an approach consistent with central monitoring and 26% (101/386) described a mixed approach. The monitoring approach was classified as ‘unspecified’ for 14% (54/386) RCTs due to a lack of sufficient detail for classification. In these cases, the monitoring approach is unclear and may have been solely on-site, solely central or a mixture of the two approaches. Two further trials reported only that on-site monitoring was not thought to be needed, giving no further details of their approach (0.5%, 2/386), meaning that they may have carried out central monitoring, or no monitoring, but this cannot be confirmed from the paper (these two trials could therefore not be classified under central monitoring, on-site monitoring, a mixed approach or be classed as ‘unspecified’). Table 1 presents the reporting patterns in each journal. In particular, among all of the journals, protocol papers in *BMJ Open* most often reported on-site monitoring (26%, 19/74); protocol papers in *JMIR Research Protocols* most often reported central monitoring (75%, 9/12); protocol papers in *Trials* most often reported a mixed approach (38%, 65/169) and protocol papers in *Medicine (Baltimore)* most often reported an unspecified approach (23%, 10/43). Only 1% (5/386) protocol papers made monitoring plans available for reference: 1 of 77 trials for on-site monitoring, 1 of 152 trials for central monitoring and 3 of 101 trials for mixed approach.

### Reporting of risk assessment

Of the 386 RCT protocol papers reporting monitoring, only 5% (19) trials explicitly mentioned risk assessments. Of the 79 RCTs reporting on whether they conducted on-site monitoring (77 that they carried it out, 2 that they did not), 6% (5) trials mentioned risk level as the justification. Of the 152 RCTs reporting central monitoring, no trial mentioned risk assessments in justifying why they only used central monitoring. Of the 101 RCTs reporting a mixed approach, 13% (13) trials explicitly referred to ‘risk-based monitoring’, ‘risk-adapted monitoring’ or ‘risk assessment’. Of the 54 RCTs whose approach was ‘unspecified’, only 1 trial (2%) stated that they had conducted the risk assessment to set out the monitoring plan.

### Reporting extent

Complete reporting of the monitoring scope, frequency and details of the organisation carrying out the monitoring was scarce. As shown in Table 2, only 8% (30/386) of RCT protocol papers completely specified the details of monitoring scope, frequency and organisation, with these details being reported most often by trials using on-site monitoring (22%, 17/77). Monitoring frequency was the least reported of the three variables. RCTs often described that they carried out monitoring ‘periodically’ or ‘regularly’ instead of giving specific intervals. Fewer details were given about monitoring scope and frequency in RCTs reporting central monitoring components than in RCTs reporting on-site monitoring components. Of the 222 RCTs that reported the monitoring organisation, more than 70% reported it to be the sponsor (Supplementary file 4).

**Table 2.** Proportion of protocol papers reporting monitoring scope, frequency and organisation by monitoring approach.

Monitoring approach^a^	Item	% Specified (n)	% Missing (n)
On-site	Scope	84 (65)	16 (12)
(N = 77)	Frequency	51 (39)	49 (38)
	Organisation	56 (43)	44 (34)
	Complete reporting	**22 (17)**	**78 (60)**
Central	Scope	35 (53)	65 (99)
(N = 152)	Frequency	25 (38)	75 (114)
	Organisation	45 (68)	55 (84)
	Complete reporting	**6 (9)**	**94 (143)**
Mixed	On-site scope	73 (74)	27 (27)
(N = 101)	On-site frequency	44 (44)	56 (57)
	Central scope	27 (27)	73 (74)
	Central frequency	25 (25)	75 (76)
	Organisation	74 (75)	26 (26)
	Complete reporting	**3 (3)**	**97 (98)**
Unspecified	Scope	59 (32)	41 (22)
(N = 54)	Frequency	7 (4)	93 (50)
	Organisation	67 (36)	33 (18)
	Complete reporting	**2 (1)**	**98 (53)**
Total	Complete reporting^a^	**8 (30)**	**92 (354)**
(N = 384^b^)			

a Complete reporting refers to where the monitoring scope, frequency and organisation were all well reported.

b A further two protocol papers reported that it was anticipated that no on-site monitoring would be needed.

### Reporting of monitoring scope

Table 3 compares characteristics of the reported monitoring scope. Source data verification (72%, 47/65), checking informed consent forms and consenting process (35%, 23/65), and ensuring protocol compliance (34%, 22/65) were the most reported items in trials conducting only on-site monitoring. The most reported items in central monitoring were checking audio records of a behavioural intervention (53%, 28/53), recruitment rate (24%, 13/53) and adverse event or serious adverse event rate (19%, 10/53). In a mixed approach, the most reported items during on-site visits were source data verification (72%, 53/74), protocol compliance (31%, 23/74) and checking the trial complied with standard operating procedures, GCP and regulatory requirements (23%, 17/74). In the central monitoring element of a mixed approach, the most reported items were recruitment rate (33%, 9/27), adverse event or serious adverse event rate (33%, 9/27) and intervention adherence (30%, 8/27). For the trials whose approach was unspecified, the monitoring scope was reported ambiguously; nevertheless, monitoring to ensure trial procedures followed protocols and applicable requirements was found in 78% of these trials’ protocol papers (25/32) and to promote data quality in 25% (8/32).

**Table 3.** Reported monitoring scope details by monitoring approach.

Monitoring approach	Monitoring scope	% Frequency (n)
On-site	Source data verification	72 (47)
(N = 65)	Informed consent form	35 (23)
	Protocol compliance	34 (22)
	Standard operating procedure/ GCP/ regulatory	31 (20)
	Adverse event/ serious adverse event rate	22 (14)
	Investigator site file	20 (13)
	Eligibility criteria	18 (12)
	Intervention	14 (9)
	Training	9 (6)
	Other	9 (6)
Central	Intervention	53 (28)
(N = 53)	Recruitment rate	24 (13)
	Adverse event/ serious adverse event rate	19 (10)
	Case report form/query return rate	13 (7)
	Other	13 (7)
	Protocol compliance	8 (4)
	Informed consent form	2 (1)
Mixed approach		
On-site	Source data verification	72 (53)
(N = 74)	Protocol compliance	31 (23)
	Standard operating procedure/ GCP/ regulatory	23 (17)
	Informed consent form	18 (13)
	Training	18 (13)
	Intervention	15 (11)
	Adverse event/ serious adverse event	12 (9)
	Investigator site file	11 (8)
	Eligibility criteria	9 (7)
	Other	8 (6)
Central	Recruitment rate	33 (9)
(N = 27)	Adverse event/ serious adverse event rate	33 (9)
	Intervention	30 (8)
	Protocol compliance	26 (7)
	Other	22 (6)
	Case report form/ query return rate	19 (5)
	Informed consent form	11 (3)
Unspecified	Procedure-related	78 (25)
(N = 32)	Data quality-related	25 (8)
	Recruitment-related	22 (7)
	Other	22 (7)
	Safety-related	13 (4)

GCP: good clinical practice.

### Reporting of monitoring frequency

**Figure 2.**
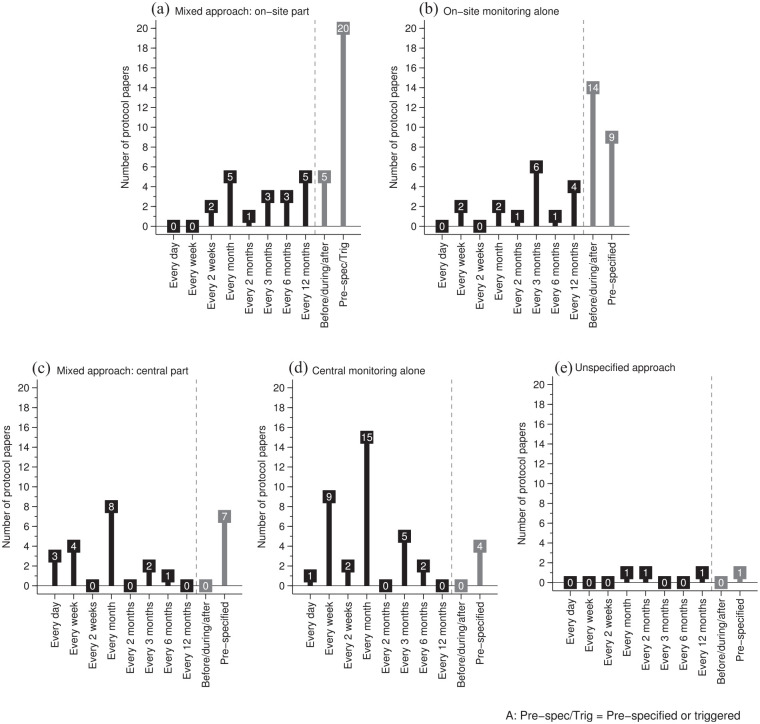
Reported monitoring frequency between on-site monitoring alone, central monitoring alone and a mixed approach.

Figure 2 illustrates the patterns of reported monitoring frequency. Where protocol papers reported on-site monitoring alone, site visits were most often reported as carried out before, during and after the trial (36%, 14/39), followed by at pre-specified time points (23%, 9/39) and quarterly (15%, 6/39). In central monitoring alone, central monitoring was reported as mostly conducted monthly (39%, 15/38) followed by weekly (24%, 9/38) and quarterly (13%, 5/38). In a mixed approach, site visits were reported mostly as carried out at pre-specified time points and/or as triggered visits (45%, 20/44). The frequency categories ‘Before, during, and after the trial’, ‘monthly’ and ‘annually’ were all equally reported (11% each, 5/44). In the central monitoring part of a mixed approach, monthly (32%, 8/25) was reported most commonly, followed by pre-specified timepoints (28%, 7/25) and weekly (16%, 4/25). Where the approach was unspecified, only four RCTs reported frequency, specifying they were carrying out monitoring twice, monthly, every 2 months or, annually.

### Replacing monitoring with ‘auditing’

We noted that a proportion of protocol papers used the term ‘audit’ to describe monitoring, possibly because the word is still being used in the SPIRIT Statement.^22^ Of the 386 RCTs reporting monitoring approaches, 6% (25) trials were identified using the term audit: 5% (4/77) trials reporting on-site monitoring, 6% (9/152) trials reporting central monitoring, 10% (10/101) trials reporting mixed approach and 4% (2/54) trials reporting the unspecified approach.

## Discussion

### Lack of transparent reporting of monitoring in protocol papers

This review shows that the level of reporting of monitoring approaches in published protocol papers is low: only around one half (48%) of protocol papers published in eight prominent journals early in 2020 reported monitoring information; the proportion of protocol papers reporting on monitoring varied widely between journals. The SPIRIT checklist^20^ explicitly requests protocols to include ‘Methods: Monitoring’ in their contents. We reviewed protocol papers which are necessarily abridged, but accessible, versions of the trial protocol, but the majority of the journals we chose comply with the SPIRIT checklist, whether through endorsement, or recommendation. Among journals’ submission guidelines, the SPIRIT checklist is endorsed or recommended in *Trials* and *Medicine (Baltimore), BMJ Open*, *JMIR Research Protocols*, *PLOS ONE* and *BMC* group journals.^27,28,31–34^ Neither *Contemporary Clinical Trials* nor *Contemporary Clinical Trials Communications* mentions it. Journals endorsing or recommending use of the SPIRIT checklist did not see greater reporting of clinical trial monitoring, indicating that they are nevertheless not systematically acting on this endorsement or recommendation in their editorial processes. However, we believe that authors of protocol papers should discuss monitoring regardless of whether the SPIRIT guidelines have been explicitly endorsed by the journal.

*Trials’* protocol papers were the most likely to report monitoring information (at 55%) among these journals, which may be explained by *Trials* having implemented a structured study protocol paper template with SPIRIT headings and item identifiers embedded for submission. A further influence on the proportion, in a journal, of protocol papers reporting monitoring information may be the types of interventions assessed in the trial protocols that journal publishes. We conducted a follow-up review of the types of intervention detailed in each of the 386 RCT protocol papers and found that clinical trials of medicinal products varied from 0% (0/57) protocol papers in JMIR Research Protocols to 18% (54/305) in Trials. Yet, we anticipate that trials of medicinal products may be underrepresented in this corpus of protocol papers. RCTs investigating medicinal products are commonly associated with higher risks than those investigating, say, behavioural interventions^35^ and are regulated differently because of this; therefore, reporting monitoring in protocol papers involving medicinal products could be regarded as more necessary. All protocol papers need to describe monitoring so that trial results can be interpreted. This may mean that journal editors need to increase word count limits to allow protocol papers to include this information.

The proportion of protocol papers reporting central monitoring alone (40%) was higher than anticipated and far higher than that observed by previous surveys. For example, Morrison et al.’s^25^ 2009 survey of a broad group of public and private sponsors of clinical research found 1 in 51 organisations reported using central monitoring alone, and Love et al.’s^24^ survey of UKCRC-registered clinical trials units in 2018 found 2 in 36 using only central monitoring. One explanation may be that not all monitoring elements were transparently reported in the protocol papers. Furthermore, 14% of protocol papers that reported monitoring information were difficult to classify into either on-site, central or a mixed approach. We encourage authors to explicitly report each trial’s monitoring approach with sufficient detail.

The proportion of protocol papers with missing monitoring details was high, especially for the monitoring frequency which was frequently missing across all monitoring approaches. Only 8% of RCTs reporting monitoring approaches specified complete details about the monitoring scope, frequency and organisation. Monitoring scope was more frequently reported for on-site monitoring than for central monitoring. This difference might imply that on-site monitoring is easier to describe, or people are less sure of how to report central monitoring.

### The mixed approach and risk-based monitoring

We expected RCTs reporting the use of a mixed approach would potentially be implementing risk-based monitoring and would therefore be more likely to give a detailed account of monitoring scope and which activities would be carried out centrally and on-site. However, monitoring scope was no better reported than was the scope for on-site or central monitoring alone. RCTs reporting a mixed approach focused more on study procedures and training and less on study documents during on-site visits than did trials reporting on-site monitoring alone, although a similar proportion in each reported source data verification and monitoring of protocol compliance (Table 3). Even though people rarely reported monitoring metrics in protocol papers, reporting monitoring on the recruitment rate, adverse event/ serious adverse event rate and protocol compliance during central monitoring broadly supported the finding of common monitoring metrics from monitoring surveys.^24,36^ In terms of the reported frequency, the mixed approach did not seem to reduce the rate of on-site visits compared with on-site monitoring alone. The frequency in central monitoring alone appeared to be higher than for the mixed approach. These findings are limited by the considerable missing information.

The chosen monitoring approaches should be proportional to the risks identified for a trial. Overall, the proportion using discussions of risk assessments or trial risk levels to justify monitoring approaches was very low (5%). This finding is contrary to the most recent monitoring surveys, which have suggested that the predefined risk level has become an essential consideration when deciding monitoring approaches.^24,36^

### Auditing is misleading in describing monitoring

Auditing is an independent quality assurance tool to ensure monitoring has been carried out effectively and follows the monitoring plan and applicable requirements to protect participant safety and data integrity.^2,3^ Both monitoring and auditing are quality assurance tools that may be used during the trial but they do not refer to the same activity. One important finding was that a small proportion (6%) of RCTs used the term ‘audit’ to describe monitoring activities. Macefield et al.^37^ also found ‘monitoring’ and ‘auditing’ were used interchangeably in describing on-site monitoring in many publications. It is notable that the SPIRIT Statement itself uses the word ‘audit’ when describing both auditing and monitoring activities.^20,22^ These results, therefore, need to be interpreted with caution because the term ‘audit’ may describe monitoring or refer to auditing itself. Elsewhere, data cleaning is often confused with monitoring.^38^

### Lack of structured language for describing monitoring

Most protocol papers in this review did not provide a dedicated, structured paragraph to describe monitoring and this might jeopardise readability. The example given below shows a reasonable succinct description of monitoring.

### Study monitoring

Proper conduct of data collection in the trial will be monitored via on-site visits of a monitoring staff member throughout the study; quality of data collected will further be monitored regularly by a statistical supervision team. After the first five participants per site are included, the quality of data (on item/trial level) per participant will be checked, aiming at detecting any error that may occur in the beginning of the project and to prevent these errors from recurring. After including participant numbers 5 to 10, the completeness and accuracy of the data on summary/scale level will be checked for all participants. Thereafter, data on a summary/scale level will be checked randomly for 1 in 5 participants.^39^

We call for all protocol papers to include a more transparent, complete and dedicated paragraph describing monitoring. Recommendations for a good description are given in Table 4 (point 4).

**Table 4.** The authors’ recommendations to improve reporting of clinical trial monitoring.

1. Clearer guidance in the ‘Methods: Monitoring’ section of the SPIRIT checklist may improve reporting (frequency, scope, approach and justification of approach) in protocols
2. Publishing a protocol paper should be the default position for all clinical trials protocols
3. Journal templates for protocol papers should include a dedicated subsection in which to present monitoring data
4. Protocol papers should provide a good description of clinical trial monitoring by the following: a. Explaining whether there will be any clinical trial monitoring and giving justification where monitoring is not needed b. Explaining how the monitoring plan was devised, for example, how the risk assessment informs the approach to monitoring c. Explaining how the monitoring will be carried out (the approach) – use terms to specify the approach, such as ‘on-site monitoring’, or ‘central monitoring’ where applicable, and keep their descriptions separate so that it is clear which elements of monitoring are being discussed d. Explaining who will be responsible (e.g. sponsor, contract research organisation, trials unit, funder) e. Explaining the scope of the monitoring within on-site and central monitoring (e.g. source data verification, checks of informed consent, adverse events) f. Explaining the frequency of each aspect of the monitoring and whether or how this will be expected to vary throughout the trial (e.g. based on accrual, site performance)
5. Monitoring should be a reserved term and should not be substituted by auditing or data cleaning
6. Documentation of monitoring approaches with sharing of monitoring findings will support efforts to determine further evidence-based approaches to monitoring

SPIRIT: Standard Protocol Items: Recommendations for Interventional Trials.

### Limitations

It is possible that the restricted choice of journals from which we took protocol papers might have led to the exclusion of publications from certain groups or of certain types of RCT. However, we believe that the journals selected represent the key journals currently publishing protocol papers for RCTs. The time frame chosen for the study was deliberately short, a snapshot of current practice. A snapshot cannot allow assessment of changes over time; only a review over a longer period could explore any impact of increasing awareness and uptake of the SPIRIT Statement.

Many of the protocol papers we reviewed included ambiguous monitoring information. Where protocol papers used the term ‘all data’ in relation to the scope of monitoring we deemed it to be too vague, and therefore omitted to include it in our analysis of the reporting of monitoring scope; the phrases ‘in real time’ and ‘throughout the trial’ were also excluded for similar reasons when analysing the reporting of monitoring frequency. However, we recognise that the reporting of monitoring scope and frequency might be underestimated because of this.

The findings of this review were restricted to the reporting of monitoring in trials for which people have chosen to publish protocol papers. Protocol papers are published for only a minority of trials. It is possible that certain types of trial, including Clinical Trials of an Investigational Medicinal Product and industry-led trials are underrepresented in the literature. The World Health Organisation International Clinical Trials Registry Platform identifies 28,763 trials as being first registered over the same dates 1 year earlier (1 January 2019 to 31 May 2019), so it is highly likely that fewer than 5% of trials have a published protocol paper.

The start of the COVID-19 pandemic made 2020 an unusual year. Publications emerging early in 2020 will reflect submissions many months earlier so the selection of trials in the published protocol papers should not have been impacted by the pandemic.

### Our recommendations

Based on our findings in this review and our trials experience, we set out in Table 4 some simple steps which we believe would improve the reporting of clinical trial monitoring in protocol papers.

## Conclusion

The SPIRIT Statement has made monitoring an essential reportable item in protocols, and efforts are being made for adherence to it in publications. However, our review concludes that the reporting of trial monitoring approaches remains suboptimal among those journals that commonly publish protocol papers, with fewer than half reporting on monitoring. This lack of transparent and structural reporting of monitoring jeopardises the clinical and trial community’s ability to judge the suitability of the chosen monitoring approaches, thereby compromising the value of protocol papers, and the credibility of the trials themselves. The results from this study should be of concern to both communities. In light of the critical role of monitoring in ensuring the quality and credibility of clinical trials, reporting it is essential. Efforts to promote the transparent reporting of monitoring will support research to develop a stronger evidence base to underpin monitoring choices and help ensure that trials are conducted well and safely.

## Supplemental Material

sj-docx-1-ctj-10.1177_17407745221143449 – Supplemental material for Lack of transparent reporting of trial monitoring approaches in randomised controlled trials: A systematic review of contemporary protocol papers Supplemental material, sj-docx-1-ctj-10.1177_17407745221143449 for Lack of transparent reporting of trial monitoring approaches in randomised controlled trials: A systematic review of contemporary protocol papers by Shao-Fan Hsieh, Victoria Yorke-Edwards, Macey L Murray, Carlos Diaz-Montana, Sharon B Love and Matthew R Sydes in Clinical Trials

sj-docx-2-ctj-10.1177_17407745221143449 – Supplemental material for Lack of transparent reporting of trial monitoring approaches in randomised controlled trials: A systematic review of contemporary protocol papers Supplemental material, sj-docx-2-ctj-10.1177_17407745221143449 for Lack of transparent reporting of trial monitoring approaches in randomised controlled trials: A systematic review of contemporary protocol papers by Shao-Fan Hsieh, Victoria Yorke-Edwards, Macey L Murray, Carlos Diaz-Montana, Sharon B Love and Matthew R Sydes in Clinical Trials

sj-docx-3-ctj-10.1177_17407745221143449 – Supplemental material for Lack of transparent reporting of trial monitoring approaches in randomised controlled trials: A systematic review of contemporary protocol papers Supplemental material, sj-docx-3-ctj-10.1177_17407745221143449 for Lack of transparent reporting of trial monitoring approaches in randomised controlled trials: A systematic review of contemporary protocol papers by Shao-Fan Hsieh, Victoria Yorke-Edwards, Macey L Murray, Carlos Diaz-Montana, Sharon B Love and Matthew R Sydes in Clinical Trials

sj-docx-4-ctj-10.1177_17407745221143449 – Supplemental material for Lack of transparent reporting of trial monitoring approaches in randomised controlled trials: A systematic review of contemporary protocol papers Supplemental material, sj-docx-4-ctj-10.1177_17407745221143449 for Lack of transparent reporting of trial monitoring approaches in randomised controlled trials: A systematic review of contemporary protocol papers by Shao-Fan Hsieh, Victoria Yorke-Edwards, Macey L Murray, Carlos Diaz-Montana, Sharon B Love and Matthew R Sydes in Clinical Trials

sj-pdf-5-ctj-10.1177_17407745221143449 – Supplemental material for Lack of transparent reporting of trial monitoring approaches in randomised controlled trials: A systematic review of contemporary protocol papers Supplemental material, sj-pdf-5-ctj-10.1177_17407745221143449 for Lack of transparent reporting of trial monitoring approaches in randomised controlled trials: A systematic review of contemporary protocol papers by Shao-Fan Hsieh, Victoria Yorke-Edwards, Macey L Murray, Carlos Diaz-Montana, Sharon B Love and Matthew R Sydes in Clinical Trials
