# Supplementary material for: Lack of transparent reporting of trial monitoring approaches in randomised controlled trials: A systematic review of contemporary protocol papers
Source: Clin Trials. 2023 Jan 11;20(2):121–32. doi: 10.1177/17407745221143449 (PMC10021127; doi:10.1177/17407745221143449)
Supplement: sj-docx-1-ctj-10.1177_17407745221143449 – Supplemental material for Lack of transparent reporting of trial monitoring approaches in randomised controlled trials: A systematic review of contemporary protocol papers [file sj-docx-1-ctj-10.1177_17407745221143449.docx]

| **PubMed search strategy:**   1. “protocol” AND “(randomized OR randomised)” 2. 1 filtered by published date, from January 01 to May 31, 2020 3. 2 and (“BMJ Open” [Journal]) 4. 2 and (“Clinical Trials” [Journal]) 5. 2 and (“Contemporary Clinical Trials” [Journal]) 6. 2 and (“Contemporary Clinical Trials Communications” [Journal]) 7. 2 and (“JMIR Research Protocols” [Journal]) 8. 2 and (“Medicine” [Journal]) 9. 2 and (“PLOS ONE” [Journal]) 10. 2 and (“Trials” [Journal]) 11. 2 and BMC *^a^* |
| --- |

^a^ The search term “all field” instead of “journal” was applied to BMC because all BMC-series journals were considered eligible for screening. The search yielded several non-BMC-series journals that were further excluded from the records.
