# Supplementary material for: Lack of transparent reporting of trial monitoring approaches in randomised controlled trials: A systematic review of contemporary protocol papers
Source: Clin Trials. 2023 Jan 11;20(2):121–32. doi: 10.1177/17407745221143449 (PMC10021127; doi:10.1177/17407745221143449)
Supplement: sj-docx-2-ctj-10.1177_17407745221143449 – Supplemental material for Lack of transparent reporting of trial monitoring approaches in randomised controlled trials: A systematic review of contemporary protocol papers [file sj-docx-2-ctj-10.1177_17407745221143449.docx]

**List of example classifications of monitoring approach**

| Monitoring approach | Example wordings |
| --- | --- |
| On-site | “A monitor will conduct monitoring visits once every 3 months.” (Su, S. et al., 2020)  “Monitoring will occur with a frequency of one visit per year per center, in which the following items will be checked: informed consents, availability of data in Trial Master File and Investigator Site Files, inclusion and exclusion criteria, SAEs, and source data verification.” (Kalkdijk-Dijkstra, A.J. et al., 2020)  “The CRA in each hospital will manage the data and monitor trial progress according to the protocol by checking the informed consent and case report forms (CRFs) and the original results of the laboratory tests and imaging studies for each participant.”  (Deng, C. et al., 2020) |
| Central | “An inspector will review the incoming data monthly and generate a data query if necessary. The inspector will review whether each electronic case report form is completed accurately. All discrepancies in the electronic case report form will be corrected by the investigator or authorized personnel in an appropriate manner.” (Wu, S. et al., 2020)  “Data integrity will be enforced through a variety of mechanisms including data rules, range checks and consistency checks against data already stored in the database.” (C. B. Miller, J. G. et al., 2020)  “Data will be collected using paper case report forms and entered into a validated trial database by the CTRU, where data quality will be monitored. Automatic and manual validation of entered data will be conducted. Data items relating to the safety and rights of individual participants will be dealt with as a priority. Data items required for the primary endpoint analysis will be manually checked at the CTRU. Missing data will be chased until it is either received or confirmed as not available at the trial analysis stage.” (Craig, Z. et al., 2020) |
| mixed | “Legally required monitoring visits will take place four times a year and according to the risk-based approach, whenever needed in between. Monitoring will be performed by the Clinical Trial Center of Medical University Innsbruck. Data checks are performed constantly by the monitoring team. Plausibility checks will be performed before the analyses.” (Pölzl, L. et al., 2020)  “The authorized person of the SMO visits before and every year after starting the trial to review protocol compliance, conduct source-data verification, assess laboratory procedures, and ensure that the study is being conducted in accordance with the protocol requirements. All adverse events (AEs) that occur during the trial will be recorded on a case report form and reviewed as part of the central data monitoring.” (Haraguchi, A. et al., 2020)  “If necessary, on-site monitoring visits can be triggered and will be carried out by either the LCTC or the sponsor representative. Data will be centrally monitored by the LCTC to promote data quality.” (Rawlinson, R. et al., 2020) |
| Unspecified | “The study will be monitored by the clinical trials center of the UZ Leuven.” (Devroe, S. et al., 2020)  “A qualified clinical trial specialist will be invited to monitor the RCT.” (Wang, C. et al., 2020)  “During the study, periodic monitoring is conducted to ensure that the protocol and International Conference on Harmonization-Good Clinical Practice (ICH-GCP) principles are being followed.” (Nabisere, R. et al., 2020) |
