# Supplementary material for: Lack of transparent reporting of trial monitoring approaches in randomised controlled trials: A systematic review of contemporary protocol papers
Source: Clin Trials. 2023 Jan 11;20(2):121–32. doi: 10.1177/17407745221143449 (PMC10021127; doi:10.1177/17407745221143449)
Supplement: sj-docx-4-ctj-10.1177_17407745221143449 – Supplemental material for Lack of transparent reporting of trial monitoring approaches in randomised controlled trials: A systematic review of contemporary protocol papers [file sj-docx-4-ctj-10.1177_17407745221143449.docx]

**Reported monitoring organisation by monitoring approach**

| Monitoring approach | Monitoring organisation | % Frequency (n) |
| --- | --- | --- |
| On-site | Sponsor | 75 (32) |
| (N=43) | Contract Research Organisation | 9 (4) |
|  | Funder | 9 (4) |
|  | Academic research institute | 5 (2) |
|  | Non-profit organisation | 2 (1) |
| Central | Sponsor | 78 (53) |
| (N=68) | Academic research institute | 15 (10) |
|  | Contract Research Organisation | 6 (4) |
|  | Funder | 1 (1) |
| Mixed approach | Sponsor | 75 (56) |
| (N=75) | Contract Research Organisation | 12 (9) |
|  | Academic research institute | 12 (9) |
|  | Non-profit organisation | 1 (1) |
| Unspecified | Sponsor | 72 (26) |
| (N=36) | Contract Research Organisation | 14 (5) |
|  | Academic research institute | 8 (3) |
|  | Funder | 3 (1) |
|  | Non-profit organisation | 3 (1) |
