# Supplementary material for: Lack of transparent reporting of trial monitoring approaches in randomised controlled trials: A systematic review of contemporary protocol papers
Source: Clin Trials. 2023 Jan 11;20(2):121–32. doi: 10.1177/17407745221143449 (PMC10021127; doi:10.1177/17407745221143449)
Supplement: sj-pdf-5-ctj-10.1177_17407745221143449 – Supplemental material for Lack of transparent reporting of trial monitoring approaches in randomised controlled trials: A systematic review of contemporary protocol papers [file sj-pdf-5-ctj-10.1177_17407745221143449.pdf]

Supplementary File 5: Data Extraction Form

journal	doi	monitoring_organisation	monitoring_approach	monitoring_scope_frequency	onsite monitoring elements	central monitoring elements	unspecified monitoring elements
BMJ Open<br>Contemporary Clinical Trials<br>Contemporary Clinical Trials Communications<br>JMIR Research Protocols<br>Medicine (Baltimore)<br>PLOS ONE<br>Trials<br>BMC group		Sponsor<br>CRO<br>Funder<br>Academic research institute<br>Non-profit organisation	on-site monitoring alone<br>central monitoring alone<br>mixed approach<br>unspecified approach	on-site scope:<br>on-site frequency:<br><br>central scope:<br>central frequency:			
